# Investigation into the annotation of protocol sequencing steps in the sequence read archive

**DOI:** 10.1186/s13742-015-0064-7

**Published:** 2015-05-09

**Authors:** Jamie Alnasir, Hugh P Shanahan

**Affiliations:** Department of Computer Science, Royal Holloway, University of London, Egham, TW20 0EX UK

**Keywords:** Next-generation sequencing, Ligation, Fragmentation, Enrichment, Protocol, Metadata, Experiment, Annotation

## Abstract

**Background:**

The workflow for the production of high-throughput sequencing data from nucleic acid samples is complex. There are a series of protocol steps to be followed in the preparation of samples for next-generation sequencing. The quantification of bias in a number of protocol steps, namely DNA fractionation, blunting, phosphorylation, adapter ligation and library enrichment, remains to be determined.

**Results:**

We examined the experimental metadata of the public repository Sequence Read Archive (SRA) in order to ascertain the level of annotation of important sequencing steps in submissions to the database. Using SQL relational database queries (using the SRAdb SQLite database generated by the Bioconductor consortium) to search for keywords commonly occurring in key preparatory protocol steps partitioned over studies, we found that 7.10%, 5.84% and 7.57% of all records (fragmentation, ligation and enrichment, respectively), had at least one keyword corresponding to one of the three protocol steps. Only 4.06% of all records, partitioned over studies, had keywords for all three steps in the protocol (5.58% of all SRA records).

**Conclusions:**

The current level of annotation in the SRA inhibits systematic studies of bias due to these protocol steps. Downstream from this, meta-analyses and comparative studies based on these data will have a source of bias that cannot be quantified at present.

**Electronic supplementary material:**

The online version of this article (doi:10.1186/s13742-015-0064-7) contains supplementary material, which is available to authorized users.

## Background

### Bias and protocols in next-generation sequencing

The introduction of next-generation sequencing technologies has transformed the fields of genomics and transcriptomics [1,2]. Much of the raw sequence read data are being deposited in public repositories such as the Sequence Read Archive (SRA) [3], Gene Expression Omnibus (GEO) [4] and ArrayExpress [5]. To produce and sequence genetic and transcriptomic data to be deposited in a public data repository such as GEO or SRA, a sample undergoes an intricate series of chemical reactions. The data are then processed before being deposited.

**Figure 1:**
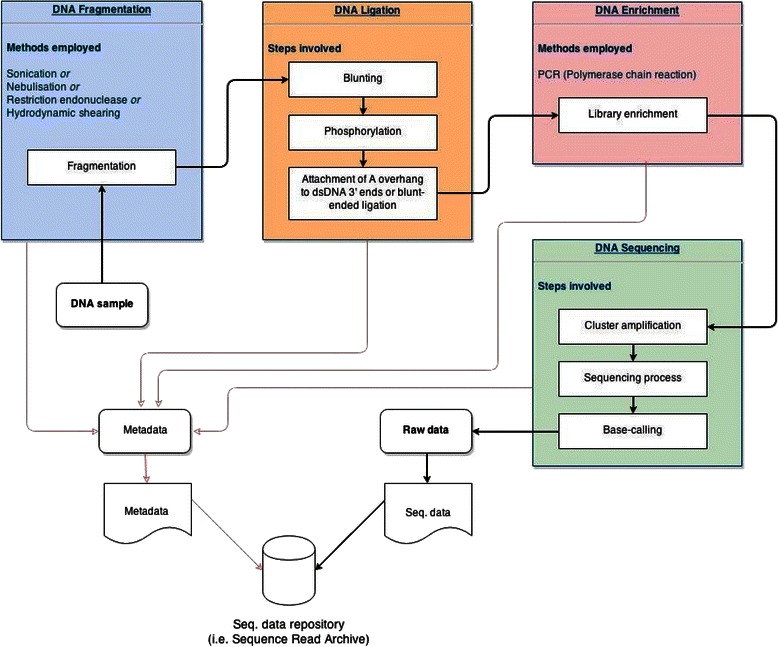
A typical next-generation sequencing workflow**.** The sequencing workflow is shown by the black arrows; red arrows depict the metadata that *should* be captured from these sequencing workflow steps. We have focused on the first three major steps.

Prior to being sequenced, a nucleic acid sample will undergo a number of steps: sample preparation, nucleic acid extraction, chemical modification (blunting, phosphorylation, ligation of instrument specific synthetic chemical sequence adapters) and chemical amplification. These steps are outlined in Figure 1. Sequencing itself is massively parallel. The results of such high-throughput next-generation sequencing workflows allow the characterisation of millions to billions of reads in a matter of days and generate large-scale data sets [1].

An extensive body of literature exists within the bioinformatics community describing the workflows used to analyse short-read data from next-generation sequencers. More than 80 papers are listed in the review of Miller, Koren and Sutton (2010) related to the assembly of sequence read data alone [6]. On the other hand, comparatively little work has been done on thoroughly determining how the protocol steps prior to sequencing can affect the final results [7]. As we will demonstrate in Next-generation sequencing protocol steps prior to sequencing section some of these steps are known to be prone to introducing bias in the sequencing data derived from the sample. This bias manifests as a deviation from the ideal uniform distribution of reads [8] and is an important factor in both genome assembly (which requires sufficient reads to form overlaps of sequences to assemble contigs) and likely impacts on expression studies that rely on the quantification of a sequence expressed (transcribed) in a sample [9]. In the following sections we discuss the various protocol steps and sources of bias they may introduce.

Bioinformatics studies seeking to characterise and model systematic errors are important, and in the case of platform specific biases, such methods can be applied in the interim before the technology is refined. To apply these methods to existing sequencing datasets, adequate metadata are required in the repository databases from which they are sourced.

An example of a systematic error that has been characterised and modelled are base call errors. According to Meacham *et al.* [9], these errors are a common feature where sequencing and next-generation sequencing technologies are used. Although these technologies have significantly reduced the costs and increased throughput associated with sequencing, they have been shown to induce more errors than preceding technologies. Focusing on the Illumina platform, with a view to demonstrating the potential impact on biological inferences, Meacham and colleagues characterised systematic errors (positional and sequence specific) that could be misinterpreted as heterozygous sites – in both individuals and SNPs – in population analyses. They found that the majority of systematic errors were sequences preceded by a G; the most common being GGT, where a T is substituted for a G. Dohm *et al.* [10] also demonstrated that wrong base calls are frequently preceded by base G, thus indicating that the Illumina base-caller software has difficulty in differentiating between GGG and GGT.

Similarly, Hansen *et al.* [11] investigated biases in RNA-seq data resulting from random hexamer priming; a method used in library preparation of dsDNA samples from RNA to be sequenced on the Illumina Genome Analyser. Their work demonstrated that random hexamer priming results in non-uniform distribution of reads resulting from positional influence on nucleotide frequencies in nucleotides up to the 13th nucleotide from the 5' end of the reads. This positional influence was reproduced across experiments, indicating the occurrence of a consistent bias. An outcome of their work is a bias count reweighting scheme, which was developed to mitigate the impact of these biases.

Finally, a relevant study on motifs that induce sequencing errors was undertaken by Allhoff *et al.* [12], who described a statistical framework for identifying sequence specific errors caused as a result of preceding bases, which they term context-specific errors (CSEs). Their method involved pooling genomic positions and screening for strand biases; a method they demonstrate to yield greater statistical power for identifying biases. Cheung *et al.* [13] studied ChIP data from Illumina’s Genome Analyser and found three types of systematic sequencing errors: those caused by GC content, mappability of sequencing reads, and regional biases that might be generated by local structure. They devised a normalisation scheme that can be applied to downstream data analyses.

A thorough understanding of the protocols that are applied prior to sequencing could provide much more subtle analyses to the ones applied above, which vary according to the pipeline of protocol steps. Furthermore, it would enable best practice guidelines for these protocols to come to the fore. In the following section we discuss the range of potential biases introduced during some of the steps occurring prior to sequencing and, while a thorough study is absent, we demonstrate evidence to suggest that some of these biases exist.

### Next-generation sequencing protocol steps prior to sequencing

The core workflow processes that are shared by next-generation sequencing technologies, and which involve protocol steps where biases may be introduced are shown in Figure 1. The protocol steps carried out before sequencing are classified into three classes: DNA fragmentation, DNA ligation and DNA enrichment. It should be noted that various protocols exist that do not require the fragmentation of DNA prior to ligation, such as PCR amplicon methods.

### DNA fragmentation

Next-generation sequencing platforms require fragmentation of double-stranded DNA (dsDNA) into sequence fragments (fragment templates or mate-pair templates) of an appropriate size, as dictated by the read-length of the platform [14]. There are currently four methodologies in use for fractionating dsDNA

- Enzymatically (with restriction endonucleases);
- Sonication [14];
- Nebulisation [15];
- Hydrodynamic shearing.

Bias induced during the fragmentation protocol step results in a large size distribution of fragment lengths.

**Enzymatic fragmentation** employs type II restriction endonucleases to cleave dsDNA at (or at close proximity to) short (3–8 bp) recognition sequence sites [16]. However, this method is known to introduce bias due to factors that might impair the activity of sequence site recognition. Kamps-Hughes *et al.* [17] utilised Illumina high-throughput sequencing to assay the enzymatic activity of type II restriction endonucleases. They examined cognate site cleavage and non-specific, non-cognate site cleavage (referred to as star activity) of restriction endonucleases (EcoRI and Mefl) by mapping millions of site-flanked reads back to the *Escherichia coli* and *Drosophila melanogaster* genomes. Their study demonstrated that, despite the high sequence specificity these enzymes exhibit, this characteristic is dependent on a number of factors such as enzyme concentration, sequence context, buffer concentration and nucleotides flanking the cleavage site.

A DNA sample may also be fractionated by **sonication**, a method in which the dsDNA is subjected to short periods of agitation by sound energy to generate fragmented DNA as a result of hydrodynamic shearing stresses [14]. Chromatin complexes of DNA and proteins have been shown to be refractory to shearing by sonication, and this results in under-representation of the sequences affected [18].

Fragmentation by **nebulisation** forces the DNA solution through a small hole. This produces a fine mist of aerosol droplets containing fractionated dsDNA in which fragment size is determined by the viscosity of the DNA solution, speed at which the solution is ejected, pressure of the gas and temperature [15]. **Hydrodynamic shearing** is a method of DNA fragmentation that involves injecting the sample solution through a narrow diameter orifice at high velocity. The resulting shearing stresses on the DNA strands cause them to break, resulting in an approximately normally distributed fragment size. Swartz and Farman investigated the effect of hydrodynamic shearing on the sequencing of telomere-associated sequences [19]. They state that searches for telomeric sequences in fungal genomic databases typically do not yield many results, and hydrodynamic shearing may be a cause of this. They found that sub-terminal regions of DNA are resistant to shearing, with breakages only occurring at the next cleavable location in relation to the terminal end of the fragments. This results in an over-representation of terminal fragments, but an under-representation of telomeric regions; as all terminal fragments are cleaved at a similar location, no contigs exist to connect the terminal and sub-terminal sequences.

### DNA ligation

#### Blunting

Unwanted 5' and 3' overhangs are removed from the double-stranded dsDNA to facilitate the ligation of platform specific synthetic DNA sequence adapters to the fragments – a process termed blunting. A number of enzymes can be utilised for this purpose such as *Klenow DNA polymerase, T4 DNA polymerase* and *mung bean nuclease*. The enzyme is used to repair the ends of the dsDNA fragments by ensuring that the ends of the complementary strands are in line with each other. Such polymerase enzymes possess 5' → 3' polymerisation activity and 3' → 5' exonuclease activity, but lack 5' → 3' exonuclease activity. The effect is that 3' overhangs on sDNA fragments are removed by the 3' → 5' exonuclease activity. The lack of 5' → 3' exonuclease activity in these enzymes means that 5' overhangs remain intact and any complementary 3' receding strands are extended and brought in line with the 5' overhung strand by the enzyme’s 5' → 3' polymerase activity. This ensures both ends are “blunted”, i.e. there is no single-stranded DNA overhang. The fidelity of polymerase enzymes used in this step is variable. Klenow polymerase has been shown to have an average error rate (mutations per base replicated) of 1.3 × 10^−4^ [18].

#### Phosphorylation

Since polymerase activity occurs in the 5' to 3' direction, it is necessary to phosphorylate the 5' ends of the blunted fragments. This can be carried out enzymatically using T4-PNK (polynucleotide kinase), which catalyses the transfer of the γ-phosphate of ATP to the 5' hydroxyl end. The efficiency of T4-PNK varies depending on the 5' nucleotide, and this can manifest itself as bias if a proportion of fragment ends remain unphosphorylated. Differences in the binding interactions between T4-PNK and the kinase substrate result in T4-PNK exhibiting bias in the preference of certain nucleotides at the first and second sequence positions of the substrate resulting in a greater activity in the phosphorylation of 5' G than for 5' C [20].

#### Attachment of a overhang to dsDNA 3' ends or blunt-ended ligation

Synthetic sequencing adapters (such as those used on the Illumina platform) normally possess a 5' T overhang to facilitate their ligation to the fragments to be sequenced. It follows that molecules in the sequencing fragment library must possess a complementary 3' A overhang; a genetically modified Klenow exo-minus is usually used to achieve this [21]. The enzyme possesses no exonuclease activity, but retains 5' → 3' polymerase activity. This is used to catalyse the attachment of A overhangs to the 3' end of the sequencing fragments so as to complement 5' T overhangs on the platform specific adapters. Alternatively, blunt-ended ligation can also be used to ligate sequence adapters with sequencing fragments.

Library preparation methods utilising DNA ligase, which is able to ligate dsDNA fragments with 5' A' overhangs to synthetic sequence adapters with 5' T overhangs, have been shown to be biased against sequences starting with T as opposed to blunt-ended ligation [22].

In their study on the impacts of Illumina sequencing bias and its implications on characterising ancient DNA, Seguin-Orlando *et al.* [23] sequenced modern DNA in parallel with different ligation strategies. In order to eliminate shearing as a source of the bias, they used sheared samples using different methods for the same ligation strategy. Their results show that the bias against sequence fragments with 5' T was unlikely to be due to the shearing method; rather it is a result of the 3' A to 5' T overhang ligation method (a primary method used by Illumina platforms). Furthermore, this correlates inversely with the concentration of sequence adapters, which is normally kept low so as to prevent hybridisation of the adapters with each other. They explain how the post-mortem degradation of ancient DNA (resulting in C deamination to U) generates misincorporation patterns in the sequencing library that can be used to recognise and characterise true ancient DNA. These patterns can also be altered during certain library construction protocol methods; Seguin-Orlando and colleagues cited the *Taq* and *Phusion* polymerase enzymes, which are integral to Illumina sequencing protocols, as a cause of this undesired modification during library preparation.

#### Adapter ligation

The ligation of synthetic dsDNA sequencing adapters (with 5' T overhang) to the fragment dsDNA (5' phosphorylated, with 3' A-overhang) again requires the use of DNA ligase, which is added in excess (concentration of 10:1) so as to ensure the attachment of as many adapters as possible per unit time. Housby *et al.* [22] point out that most studies of the DNA replication process have centred on the fidelity of DNA polymerase and the importance of understanding all the mechanisms that ensure faithful copying of DNA sequences during replication.

### DNA enrichment

In order to achieve sufficient quantities of sequencing samples for sequencing, an enrichment process must be applied to the adapter-ligated fragment library. The polymerase chain reaction (PCR) is a mainstay method in DNA enrichment [24]. It is useful for enriching the fragment library since it replicates only those fragments to which an adapter, encapsulating a PCR primer binding region, is attached. Those fragments not ligated to adapters will not be replicated by virtue of lacking the PCR primer site which is located on the ligated adapter. However, PCR amplification may introduce bias in the form of non-uniform distribution of reads; it can introduce artefacts into the library prepared for sequencing [25]. The significant variation in the fidelity of polymerase enzymes on which PCR depends has long been established [18]. There are a number of origins for such artefacts: re-arrangement of the DNA resulting in Chimera formation, formation of hetero-duplex molecules, and DNA polymerase errors. Further discussion of these is beyond the scope of this article; however, we direct the reader to a study by Acinas *et al.* [25], who looked at PCR-induced artefacts in sequencing library construction.

It has been long established that PCR is impaired by GC-bias in the fragments to be enriched [24-26]. Kozarewa *et al.* [24] demonstrated that using a PCR amplification-free enrichment step that relies solely on cluster amplification on the sequencing platform for the enrichment of the library, resulted in a more uniform distribution of reads.

Given the biases in polymerase activity [27,18], a number of commercially produced genetically modified polymerase enzymes have been developed to confer greater fidelity. An investigation by Quail *et al.* [28] compared the fidelity of two commercially available polymerase enzymes, *Kappa HiFi* and *Phusion* polymerase, against PCR-free sequencing of four genomes of varying GC content. They demonstrated variation between these two polymerases and found the profile of *Kappa-Hifi* (as depicted by plots of normalised sequencing depth vs. % coverage) to be closer to the profile seen with no amplification, as compared with *Phusion* polymerase.

DNA damage has also been shown to influence nucleotide incorporation and can introduce bias dependent on the preferences of the polymerase catalysing the reaction. Investigation into nucleotide incorporation preferences of different polymerases can be achieved using modified nucleotide 8-Oxo-7,8-dihydro-2′-deoxyguanosine (8-oxodG), which can exhibit both an *anti-conformation* (allowing normal Watson–Crick base pairing) or *syn-conformation* (that undergoes Hoogsteen bonding) [29]. Sikorsky *et al.* investigated this and describe how the ratio of dCMP to dAMP insertion, corresponding to 8-oxodG, is dependent on the class of polymerase. They found that both the fidelity and amplification efficiency of *Taq* DNA polymerase are susceptible to lesions on the fragment to be enriched.

Other enrichment methodologies can also be a source of bias; for example, multiple strand displacement amplification (MDA) can result in preferential amplification of certain sequences [30].

### Annotation of publicly deposited data

We have described the above-mentioned sequencing workflow steps that have the potential to introduce bias into the resulting DNA sequencing data, particularly focusing on sequence read DNA data and the relevant metadata. However, RNA sequencing data have also been demonstrated to be prone to biases in protocol steps. Random hexamer priming [11] is an example, though other sources may well exist. This requires similar study but is beyond the scope of this paper at this point.

There exist a number of different standards for annotating genomic and transcriptomic data sets, including Minimum Information for a Micro-array Experiment (MIAME) [31] and Minimum Information for a Sequencing Experiment (MINSEQE) [32], as well as document markup formats including Microarray Gene Expression Markup Language (MAGE-ML). Ostensibly, appropriate use of the above standards (notwithstanding the absence of an agreed vocabulary) should ensure that one can disentangle the effects of different biases. We note that SRA [3], the focus of this paper, conforms to MINSEQE requirements.

For the remainder of this paper we will explore the level of annotation of the preparatory protocols in data publicly deposited in the SRA, one the main repositories for next-generation sequencing data. In particular, we will describe in detail the SRA metadata schema and the relevant fields for this study. Having constructed a list of relevant keywords for the preparatory steps described above, we will present their relative frequency in the SRA metadata and the non-standard methods used to describe the annotation. Finally, we will discuss the implications of the low level of coverage of these keywords and the overall structure of metadata in the SRA and how this inhibits a more systematic study.

## Data description

### The sequence read archive

The SRA is one of the primary repositories for high-throughput sequencing data [3]. As of December 2013, according to our queries, there were 29,598 studies in the database. The archive is synchronised periodically as part of the International Nucleotide Sequence Database Collaboration (INSDC) and this allows data deposited to any site that is part of the collaboration to be accessed via any of the others. In addition to the raw sequence data that comprise the bulk of the total data in the archive, metadata describing experimental parameters are also stored and made accessible to users. A number of such parameters are recorded with depositions – for example the design of the experiment, details of species, cell lines, samples and identifiers for sequencing platforms, and protocols [33]. As outlined below, there is a detailed database schema for conveying the metadata associated with each deposition. Given the large number of depositions and facilities for depositing metadata, the SRA is an excellent database to examine the range of different protocols.

### SRA database schema

**Figure 2:**
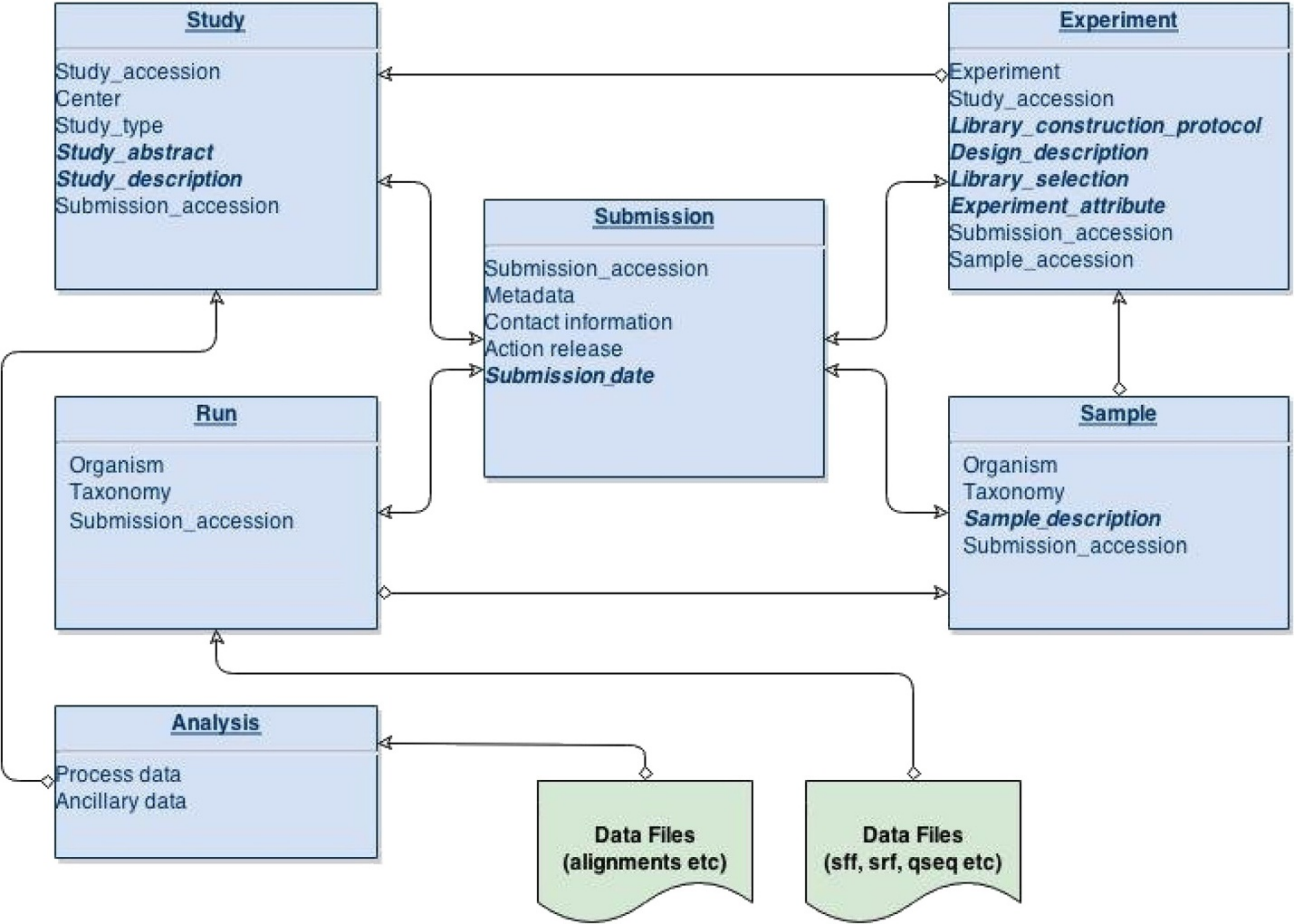
Schema diagram of the SRA relational SQLite database based on the SQL metadata. Field names in emphasis have been probed for protocol step annotation (Table 2) together with submission table date-stamp. Diamonds represent one-to-many relationships. Fields in bold emphasis are those with relevant experimental metadata.

The schema for the Bioconductor SRAdb SQLite relational database, which is derived from and reflects the underlying NCBI SRA XML data (see Methods for further details), is shown in Figure 2. This particularly focuses on the metadata for sequence read data [34,35]. The fields in bold are those relevant to this paper. SRA metadata is organised and stored in a relational database format across a number of tables: *Submission, Study, Experiment, Run, Sample and Analysis*. Each of the fields allows free-text entries.

Raw sequence data is stored under specifically named directories described in the metadata; the *Study* table is the master table in this case. For each study entry, there are many *Experiment* entries and corresponding *Sample* and *Run* entries. Given that *Experiment, Sample* and *Run* tables have many-to-one relationships to each study entry, we have aggregated them by *Study* entry.

## Analyses

### Annotation of protocols in SRA metadata

A search for the keywords listed in Table 1 was carried out over all the metadata fields (listed in Table 2) deposited in the SRA. The results are summarised in Table 3.

**Table 1:** **Table depicting the structured word list for given protocol steps (fragmentation, adapter-ligation, enrichment)**

Protocol step structured word lists		
Fragmentation	Adapter-ligation	Enrichment
*shear*	*adapter*	*clone%*
*restriction*	*ligat%*	*clonin%*
*digest*	*blunt%*	*vector%*
*fragment*	*phosphorylat%*	*pcr*
*breaks*	*overhang*	*amplif%*
*acoustic*	*t4-pnk*	*polymerase*
*nebulisation*	*t4*	*taq*
*nebulization*	*pnk*	*phusion*
*nebuliz*	*kinase*	*temperat%*
*nebulis*	*a-tail*	*thermal%*
*sonic*		*anneal%*
		*denature%*

The ‘%’ symbol denotes fuzzy-match logic, for instance *%amplif* will match (*amplify* and *amplified*).

**Table 2:** **SRA developer documentation (col_desc table found in SQLite DB)**

Table	Field	Description
Study	**study_abstract**	Briefly describes the goals, purpose and scope of the Study. This need not be listed if it can be inherited from a referenced publication.
Study	**study_description**	More extensive free-form description of the study.
Sample	**description**	Free-form text describing the sample, its origin and its method of isolation.
Experiment	**design_description**	More details about the set-up and goals of the experiment as supplied by the Investigator.
Experiment	**library_selection**	Whether any method was used to select and/or enrich the material being sequenced.
Experiment	**library_construction_protocol**	Free-form text describing the protocol by which the sequencing library was constructed.
Experiment	**experiment_attribute**	Properties and attributes of the experiment. These can be entered as free-form tag-value pairs.

**Table 3:** **Metadata SQL query results**

Table	Field	Total records (in table)	*Annotation record counts*			
			Fragmentation	Adapter ligation	Enrichment	All steps
Study	**study_abstract**	**29,598**	**376 (1.27%)**	**138 (0.47%)**	**941 (3.18%)**	**12 (0.04%)**
Study	**study_description**		**292 (0.98%)**	**136 (0.51%)**	**488 (1.65%)**	**53 (0.18%)**
Sample	**description**	**480,222**	**1,632 (0.34%)**	**896 (0.19%)**	**2159 (0.45%)**	**653 (0.14%)**
Experiment	**design_description**	**419,620**	**11,705 (2.79%)**	**6,382 (1.53%)**	**16,779 (4.00%)**	**2,691 (0.64%)**
Experiment	**library_selection**		**1,493 (0.36%)**	**0 (0%)**	**0 (0%)**	**0 (0%)**
Experiment	**library_construction_protocol**		**29,799 (7.10%)**	**24,486 (5.84%)**	**31,782 (7.57%)**	**17,021 (4.06%)**
Experiment	**experiment_attribute**		**422 (0.10%)**	**1,026 (0.24%)**	**2,814 (0.67%)**	**129 (0.03%)**

Each column (on the right side of the table dividing line) represents a sequencing step for which a word list is used to filter records where this step is annotated. Counts are the number of *experiment* records exhibiting this particular annotation. “All steps” indicates the number of fields containing all three types of protocol step annotation, i.e. they all have keywords from each of the keyword lists.

The most populated field in terms of protocol annotation was the library_construction_protocol field of the experiment table (*Experiment:library_construction_protocol).* Despite this, fragmentation, adapter ligation and enrichment were annotated in 7.10%, 5.84% and 7.57% of all records respectively, with only 4.06% of entries having all three protocol steps annotated. We also found that approximately half (212,070; 51.12%) of the total records have a null entry in the library_construction protocol field. The next most annotated field in terms of next-generation sequencing sample preparation protocol steps was the experiment table *(Experiment: design_description)*, with 2.79%, 1.53% and 4.00% being annotated for fragmentation, adapter ligation and enrichment annotation respectively, and only 0.64% of the records covering all of the three main protocol steps.

A small number of depositions have protocol information within their study abstracts and/or study descriptions. Understandably, these fields may contain words for or describe a protocol step in the abstract if it constituted a notable aspect of the submitter’s experiment; however, proper annotation should occur in the *Experiment:library_construction_protocol* field. The vast majority of the small number of entries in the *Study:study_abstract* field had corresponding entries in the correct *Experiment:library_construction_protocol* field (99.2%, 100% and 100% for fragmentation, ligation and library enrichment annotations respectively). Likewise entries in the *Experiment:design_description* field also had corresponding *Experiment:library_construction_protocol* field entries (99.0%, 100%, 100% for fragmentation, ligation and library enrichment annotations respectively).

Our analysis of SRA metadata found that only 84,911 out of a total of 414,788 experimental records (20.47%) exhibited annotation for *any* of the three protocol steps, whilst only 16,930 of the total had all three key annotation steps documented. These “fully annotated” records (those that have documented the key protocol steps of fragmentation, ligation and library enrichment) comprise only 4.06% of all the aforementioned records.

### Low level of protocol step annotation in the metadata across all top-level SRA studies

As outlined in Methods and Reagent kit data section, focusing on the *Experiment* records may not reflect the level of annotations across individual studies. In order to avoid this within our structured queries we collated by top-level study.

When examining the SRA metadata from the top-most *Study* level, the extent of annotation of the key next-generation sequencing workflow protocol steps (fragmentation, ligation and enrichment) was also found to be low. Out of 29,598 study records, 21,799 (73.6%) of the studies – where all of their associated records were associated – had no annotation whatsoever. The number of studies with full annotation was 1,409 (4.7%).

### Reagent kit data

The use of reagent kits and SRA users’ corresponding annotation was also tested; additional SQL queries were written to search for the keywords “reagent” or “kit” in the experiment table *Library_construction_protocol* field. Counts of these records were compared against those probed for *all* annotation types. The results show that there is considerable overlap between annotated fields explicitly discussing the protocol and those mentioning a reagent kit; i.e. 23,288 of all experiment level records (5.55%) annotated for *all* protocol steps also contained the keywords “reagent” or “kit”, and this partitions across 2,055 (6.94%) top-level SRA studies.

### Annotation of protocol steps in the metadata: significant variation in how this is stored at the *experiment* record level

**Figure 3:**
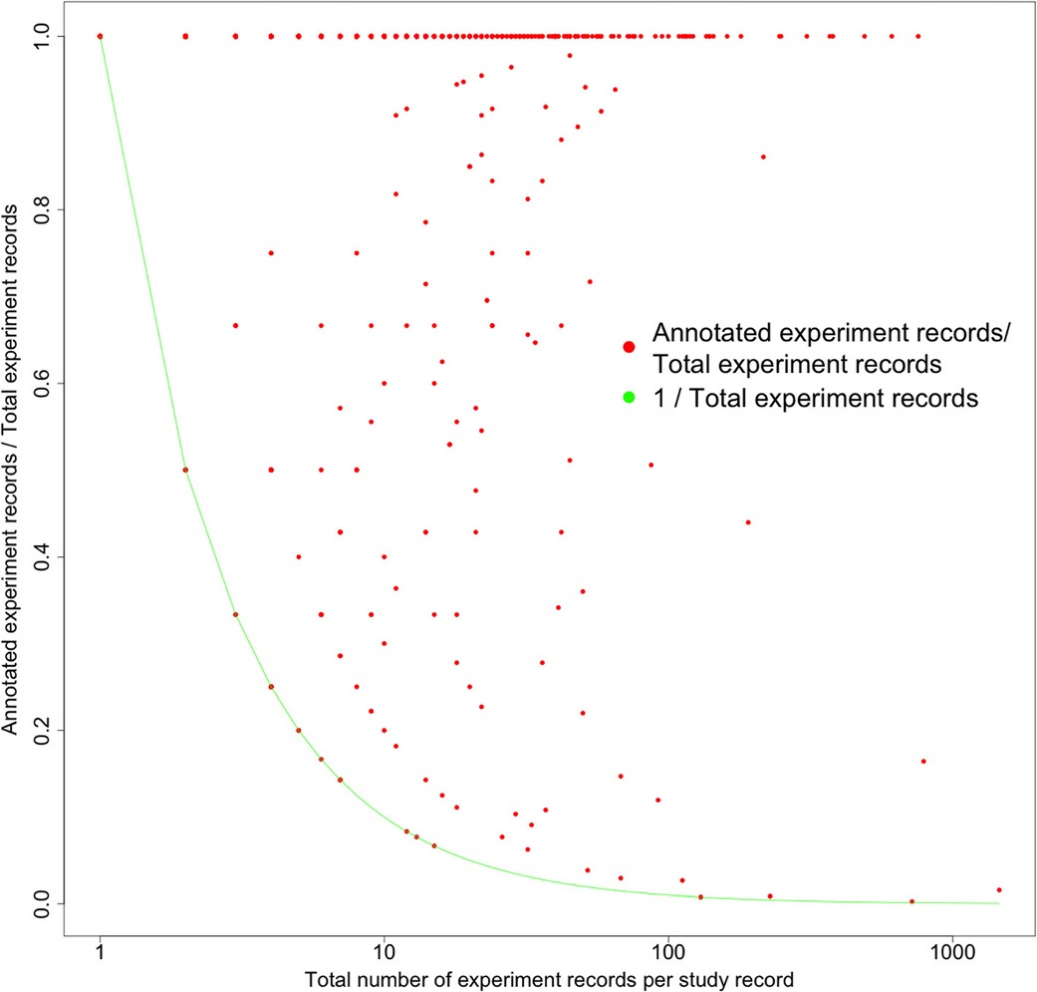
Ratio of annotated experiment records to total vs total number of experiment records per study. Only study records where at least one experiment record is fully annotated are included. Points where the ratio is 1 represent study records where all of the experiment records in a given study are fully annotated. The green line is a plot of 1/total number of experiment records in a given study. Points lying along this line are those studies where only one experiment record is fully annotated (presumably to represent the annotation of all the other experiment records). Points between these two curves represent studies where an intermediate number (neither 1 or all of the experiment records) are annotated.

We further examined the storage of annotation in the *experiment* records meeting our minimum standard (i.e. having annotations for all three protocol steps). This revealed significant variation in the metadata stored for a given study (see Figure 3). In the SRA, the number of experiment records associated with a given top-level study can vary from a single *study* with one *experiment* record, to a single *study* with 15,548 *experiment* records (i.e. there are many studies with few experiments, and few studies with many experiments). A study was non-conservatively considered as being annotated for *all three protocol steps* if *at least one* of its corresponding *experiment* records contained annotations for these three steps. The variation shows there are inconsistencies in how annotations are stored across multiple *experiment* records for a given *study*. Given the potential to store redundant metadata, it is possible that in a study containing a small number of experiment records all may be annotated, whilst in a larger *study* containing many *experiment* records only one or a select few of these *experiment* records could be annotated.

## Discussion

From a variety of articles it is clear that potential biases may exist in next-generation sequencing data due to the preparatory protocols carried out on the samples before submission. It is important to understand the size of these biases in order to determine best practices and how they can affect issues such as the validity of comparative approaches in genomics using these data sets.

With this in mind, we have carried out a thorough study of the level of annotation of the initial protocol metadata deposited at the SRA. We have shown that the amount of annotation is very sparse with around 4% of the studies having keywords corresponding to all the steps relevant to the protocols. Around half of entries for experiments have a null (empty) entry in the fields where these data should be recorded.

In addition to the poor level of annotation as previously discussed, there are further issues associated with the metadata deposited in the SRA. Depositors are unclear about whether to provide annotation for all or some of the records at the experiment record level, therefore annotation is inconsistent. This is shown by the large variance in experiment fields having annotation in any individual study. We have also found practices such as the use of URLs to provide a link to the appropriate annotation (see Additional file 1). Accessing basic data such as date stamps is very difficult and appears to be stored inconsistently.

More seriously, the use of the free text within fields means that any large-scale computational survey of these metadata, beyond the use of simple keyword searches, would require advanced text-mining techniques.

Nakazato *et al.* undertook a study to constrain sequencing data by the submission accession link cited in publications; their rationale was that this constraint would yield only higher quality submissions to the SRA [33]. Although very useful, their approach to the issue of SRA meta-data is from a different perspective and stops short of examining the metadata content in the context of the types of biases that can result due to the protocol steps in the next-generation sequencing workflow, as we have done. However, their work corroborates our view: important metadata fields are free-text fields that are not amenable to efficient comparison.

As noted previously the SRA conforms to the MINSEQE specification [32]. This stipulates five essential components for submitting a high-throughput sequencing experiment, they are i) description of the biological system, samples, and the experimental variables under study ii) sequence read data for each assay iii) ‘final’ processed (or summary) data for the set of assays in the study iv) general information about the experiment and sample-data relationships and v) Essential experimental and data processing protocols. The fifth component in particular, essential experimental and data processing protocols, concerns the discussion in this paper. Nonetheless, SRA protocol data is not amenable to automated methods due to the use of free-text fields and the absence of a more structured approach to recording important experiment protocol information. Depositors are likewise not obliged to complete this information.

Given the size of the data sets being deposited in the SRA, it is unfortunate that more strictly enforced guidelines on these metadata have not been provided, along with the use of agreed vocabularies and ontologies. A more structured approach to metadata deposition would allow a deeper analysis and hence the examination in much more detail of the source of these biases, along with the quality issue raised by Nakazato *et al*. above. These data would ideally be represented via an ontology that is tailored towards protocols, and submitters would be obliged to fill out such data. A less elaborate, though still helpful, approach would be to oblige submitters to refer to the manufacturer’s reagent kit via a specific field with fixed values. Likewise, a clearer policy on the submission of protocol data at the study and experiment level would also avoid confusion.

## Potential implications

The SRA is a huge resource of genomic and transcriptomic data; as of September 2010, more than 60 trillion base pairs were available for download [3]. In particular, this resource should be invaluable for comparative genomics and meta-analyses. However, as demonstrated there is currently a major shortfall in the level of annotation provided for key protocol steps. To enable a wider and more comprehensive use of these resources the community should engage with the SRA to provide these details. The community and archives at NCBI, EBI and DDBJ need to work together to tighten the requirements for metadata submission by making protocol steps mandatory, and through the use of controlled vocabularies.

## Methods

Metadata for sequence read DNA sequencing data in the SRA public repository [3] was acquired from the Bioconductor project in SQLite format [36]. The metadata extraction timestamp was 2013-12-03. The data set and SQL scripts supporting the results of this article are available in the *GigaDB* repository [37].

### Selecting appropriate fields for annotation of protocol steps

The SRA documentation [34] was utilised, in particular a metadata developer documentation table containing a list of fields and descriptions of the information to be stored. This table was used to determine the fields that would be most appropriate to probe for metadata annotation using the structured word lists (Table 2).

### SRA XML DTD and SRAdb SQLite differences

The SRAdb SQLite database package produced by Bioconductor was utilised for this paper. In order to ensure that the SRAdb is a good proxy for the underlying NCBI SRA XML data, all fields from both the Bioconductor SRAdb SQL schema and NCBI SRA XML fields were extracted into separate text files for each SRA table. Each field from the NCBI XSD XML schema was then tested for its presence (or absence) in the corresponding Bioconductor SRAdb SQL table.

Three fields were found in the XML data that are not mapped to SRAdb SQLite and may contain further protocol data: *Library_Descriptor*, *Sample_Attributes* and *Submission_Attribute*. However, on further inspection, these fields are either deprecated (*Library_Descriptor*), store only biological sample data (*Sample_Attributes*), or carry data about the actual submission (*Submission_Attribute*).

### Probing free-text fields for annotation of protocol keywords

In the absence of greater structure in the fields, a structured word list relevant to the fragmentation, enrichment and adapter-ligation protocol steps was constructed. This word list is shown in Table 1. The metadata table and column descriptions from the SRA developer documentation were used as a guide to select appropriate fields, and were inspected using SQL queries to quantify the number of records appearing to be annotated for a given protocol step. Occurrences in the field under inspection of one or more of the words in the list for a given protocol step were recorded. There is substantial overlap between the terms from the different lists, as shown in Additional file 1: Figure S1.

### Aggregating data over experiment records

Metadata from an *Experiment* record are directly associated with an individual set of sequence data deposited at the SRA. However, as noted in Reagent kit data section, metadata deposited in one or some subsets of *Experiment* records may in fact represent equivalent metadata for all the *Experiment* records of a given *Study*. In order to investigate this, the relevant fields of all the *Experiment* records for every given *Study* record were aggregated. Searches for the keywords outlined above were repeated. Any hits in the above lists were treated as evidence of metadata for the protocol steps for the entire study.

### Availability and requirements

- Project name: Investigation into the annotation of protocol sequencing steps in the Sequence Read Archive SQL scripts
- Project home page: https://github.com/gigascience/paper-alnasir2015
- Operating system: Any supporting SQLite3
- Programming language: SQL
- Other requirements: Bioconductor SRAdb SQLite database
- License: GPL v3.

## Availability of supporting data

The data set and SQL scripts supporting the results of this article are available in the *GigaDB* repository [37].

## Additional file

Additional file 1:
**Supplementary information.**

## Abbreviations

DDBJ: DNA data Bank of Japan
EBI: European Bioinformatics Institute
GEO: Gene Expression Omnibus
INSDC: International Nucleotide sequence database collaboration
MAGE-ML: Microarray gene expression markup language
MIAME: Minimum information for a micro-array experiment
MINSEQE: Minimum information about a high-throughput sequencing experiment
NCBI: National center for biotechnology information
SQL: Structured query language
SRA: Sequence read archive
XML: Extensible markup language
