# Supplementary material for: Investigation into the annotation of protocol sequencing steps in the sequence read archive
Source: Gigascience. 2015 May 9;4:23. doi: 10.1186/s13742-015-0064-7 (PMC4425880; doi:10.1186/s13742-015-0064-7)
Supplement: Additional file 1: — Supplementary information. [file 13742_2015_64_MOESM1_ESM.docx]

Supplementary Information

***Overlap between different protocol search terms***

The gold standard for protocol step annotation is an experiment record contain annotations for all three protocol steps. Figure S1 demonstrates a search term from one of the protocol steps is likely to overlap with search terms from other protocol steps.

**
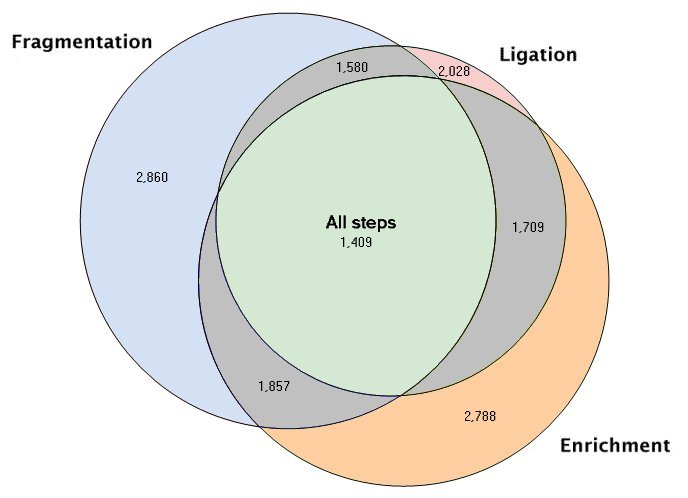
**

**Figure S1** Venn diagram depicting the coverage of annotations across the few SRA *studies* possessing at least one *experiment* record. Records sharing two annotation types are shown in grey: fragmentation and ligation (grey-top), ligation and enrichment (grey-right) and fragmentation and enrichment (grey bottom-left). *Studies* containing at least one *experiment* record with all three protocol step annotations are shown in green.

**URL as annotation**

It is worth noting that 5,616 (1.34% of total) records of the field *Experiment: library_construction_protocol* contained a URL (uniform resource locator). Whilst this is useful for external reference and in providing an audit trail in terms of protocol, the external resource referred to may be online/unavailable at a time after original submission. It is therefore necessary to ensure that a URL is only supplementary and does not constitute annotation of a protocol step in its entirety. Furthermore the use of URLs is not amenable to automated means of comparison.[31]

| **Table** | **Field** | **Total Records** | **URL Counts** |
| --- | --- | --- | --- |
| study | **study_abstract** | **29,598** | **444** |
| study | **study_description** |  | **817** |
| study | **study_attribute** |  | **422** |
| sample | **description** | **29,598** | **781** |
| sample | **sample_attribute** |  | **866** |
| experiment | **design_description** | **419,620** | **700** |
| experiment | **library_construction_protocol** |  | **5616** |
| experiment | **experiment_attribute** |  | **1,236** |

**Table S2** Counts of fields containing a URL to an external site.
